# Prenatal vitamin D supplementation mitigates inflammation-related alveolar remodeling in neonatal mice

**DOI:** 10.1152/ajplung.00367.2022

**Published:** 2023-05-31

**Authors:** Julia Waiden, Motaharehsadat Heydarian, Prajakta Oak, Markus Koschlig, Nona Kamgari, Michael Hagemann, Matthias Wjst, Anne Hilgendorff

**Affiliations:** ^1^Institute for Lung Health and Immunity and Comprehensive Pneumology Center, Helmholtz Zentrum München, German Center for Lung Research (DZL), Munich, Germany; ^2^Core Facility Laboratory Animal Services, Helmholtz Zentrum München, German Research Center for Environmental Health, Munich, Germany; ^3^Institut für KI und Informatik in der Medizin, Lehrstuhl für Medizinische Informatik, Klinikum rechts der Isar, Munich, Germany; ^4^Center for Comprehensive Developmental Care (CDeCLMU) at the Social Pediatric Center, Dr. von Hauner Children’s Hospital, LMU Hospital, Ludwig-Maximilian-University, Munich, Germany

**Keywords:** bronchopulmonary dysplasia, mechanical ventilation, oxygen toxicity, prenatal treatment, vitamin D

## Abstract

The development of chronic lung disease in the neonate, also known as bronchopulmonary dysplasia (BPD), is the most common long-term complication in prematurely born infants. In BPD, the disease-characteristic inflammatory response culminates in nonreversible remodeling of the developing gas exchange area, provoked by the impact of postnatal treatments such as mechanical ventilation (MV) and oxygen treatment. To evaluate the potential of prenatal treatment regimens to modulate this inflammatory response and thereby impact the vulnerability of the lung toward postnatal injury, we designed a multilayered preclinical mouse model. After administration of either prenatal vitamin D-enriched (VitD+; 1,500 IU/g food) or -deprived (VitD−; <10 IU/kg) food during gestation in C57B6 mice (the onset of mating until birth), neonatal mice were exposed to hyperoxia (Fi_O_2__ = 0.4) with or without MV for 8 h at *days 5–7* of life, whereas controls spontaneously breathed room air. Prenatal vitamin D supplementation resulted in a decreased number of monocytes/macrophages in the neonatal lung undergoing postnatal injury together with reduced TGF-β pathway activation. In consequence, neonatal mice that received a VitD+ diet during gestation demonstrated less extracellular matrix (ECM) remodeling upon lung injury, reflected by the reduction of pulmonary α-smooth muscle actin-positive fibroblasts, decreased collagen and elastin deposition, and lower amounts of interstitial tissue in the lung periphery. In conclusion, our findings support strategies that attempt to prevent vitamin D insufficiency during pregnancy as they could impact lung health in the offspring by mitigating inflammatory changes in neonatal lung injury and ameliorating subsequent remodeling of the developing gas exchange area.

**NEW & NOTEWORTHY** Vitamin D-enriched diet during gestation resulted in reduced lung inflammation and matrix remodeling in neonatal mice exposed to clinically relevant, postnatal injury. The results underscore the need to monitor the subclinical effects of vitamin D insufficiency that impact health in the offspring when other risk factors come into play.

## Graphical abstract

**Figure:**
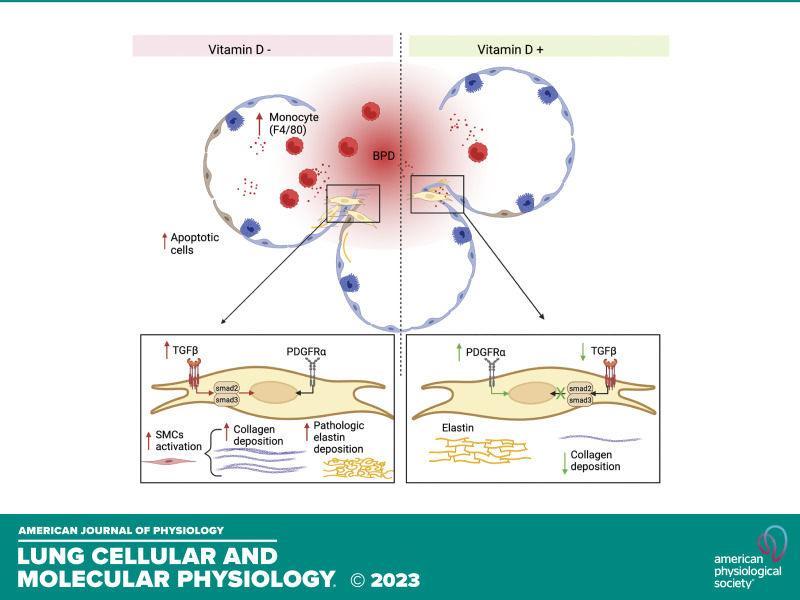

## INTRODUCTION

Pulmonary complications are the most prevalent morbidity in perinatal medicine (1), and bronchopulmonary dysplasia (BPD) as the most common form of neonatal chronic lung disease affects up to 30% of premature infants long term (2). BPD is characterized by the impaired development of the gas exchange area as a consequence of cellular malfunction and significant matrix remodeling following premature birth (3). Postnatal risk factors such as mechanical ventilation (MV) and oxygen (O_2_) toxicity have been identified to drive disease development (4), modulated by prenatal risk factors. The common pathophysiology provoked by pre- and postnatal insults is a disease-characteristic pulmonary inflammatory response (5, 6) known to drive the pathological remodeling process of the pulmonary scaffold (7, 8).

Subsequently, immune-modulatory effects of different substances applied in neonatal care gained increasing interest including surfactant, nitric oxide, vitamin A, and others (9–11). Vitamin D, essential in pre-and postnatal treatment regimens for its pivotal role in bone accretion (12–14), was demonstrated to impact the prevalence of immune-related diseases in infants including infections. Studies furthermore revealed the effects of vitamin D on the increased risk for preterm birth and the development of birth-related complications (15, 16). Regarding pulmonary morbidity, clinical observations and in vivo studies demonstrated an effect of vitamin D levels on lung function and respiratory infection rates (17, 18). However, discussions about its potential function in lung health and disease development remain controversial (19).

Reflecting on the complex nature of vitamin D-related effects, recommended doses for vitamin D supplementation vary between 400–1,000 IU/kg body weight (BW) for preterm infants (14, 20, 21). Guidelines agree, however, that prenatal supplementation is critical to prevent vitamin D deficiency. Whereas the alarming consequences of vitamin D deficiency during pregnancy (0% to 27% in pregnant women) have been studied to some extent (22), and vitamin D insufficiency is even more prevalent, ranging from 33.9% to 70.4% among pregnant women (23), but its health effects on the offspring have not been addressed in considerable detail.

We therefore opted to specifically address the most prevalent condition, i.e., vitamin D insufficiency, that most likely remains clinically silent regarding obvious signs of vitamin D deficiency. Our aim was to demonstrate that “subclinical” vitamin D insufficiency during pregnancy, i.e., withdrawal of vitamin D only from the onset of mating to birth and no ultraviolet (UV)-light restrictions, is able to impact neonatal health. We employed a multilayered preclinical model using prenatal diet regimen in combination with postnatal lung injury in otherwise healthy neonatal mice and addressed the potential of vitamin D to modulate immune-related processes and subsequent remodeling processes in the developing alveolar niche. Greater insight into the effects of such a treatment, especially regarding the vulnerability of the developing organism to (postnatal) organ injury, could inform recommendations for treatment regimen, particularly in high-risk patient populations suffering from premature birth and the subsequent immaturity of metabolism and immunity.

## MATERIALS AND METHODS

### Animal Studies

For breeding, pathogen-free male and female C57BL/6 (wildtype, WT) mice were obtained from Charles River (Sulzfeld, Germany) and housed at constant temperature and humidity, a 12-h light cycle, and food and water ad libitum as described previously (24). From the onset of mating to the end of gestation, female mice received a vitamin D3 [Calcitriol, (1,25-dihydroxy-vitamin D3)]-enriched (1,500 IU/kg) or reduced (<10 IU/kg) (ssniff-Spezialdiäten GmbH) diet adapted from previous reports (25). The food composition was identical except for vitamin D_3_ content. No UV-light restrictions were applied. After spontaneous delivery, newborn mice [3.7 ± 0.5 g; body weight (BW)] were randomly assigned to postnatal treatment on *postnatal day* (PND) *5–7*. The pups received oxygen supplementation (O_2_) [fracture of inspired oxygen (Fi_O_2__ = 0.4) with or without mechanical ventilation (MV)]; controls spontaneously breathed room air (RA) (Fi_O_2__ = 0.21) for 8 h as described previously (26). In short, mice underwent *1*) tracheotomy after ketamine (≈60 µg/g BW) and xylazine (≈12 µg/g BW) sedation for MV at 180 breaths/min (MicroVent 848; Harvard Apparatus, Holliston, MA; mimic of clinical conditions: mean tidal volume 8.68 µL/g BW; peak airway pressure 12–13 cmH_2_O, mean airway pressure 11–12 cmH_2_O) or *2*) spontaneously breathed O_2_ or RA after sham surgery under mild sedation (27). Viable pups were euthanized (sodium pentobarbital), and lungs were harvested for analysis. All animal experiments followed strict governmental and international guidelines approved by the local government for the administrative region of Upper Bavaria Animal Care and Use Committee.

### Assessment of Matrix Remodeling

Postmortem, lungs for structural analysis were immediately fixed with 4% paraformaldehyde. Hematoxylin and eosin (H&E) staining was performed on four random paraffin-embedded tissue sections (4 mice/group) to quantify total tissue area in four fields of view (FOV) per section (×200 magnification) using the Bioquant software. The relative amount of insoluble cross-linked elastin in lung tissue was assessed using Cathy Hart’s stain in four random paraffin tissue sections (5 mice/group) as described previously (28). Quantitative elastin distribution was assessed using color analysis mode (BIOQUANT Life Science 2017 v17.5.6 BIOQUANT Image Analysis Corporation, Nashville) distinguishing overall tissue and elastin in four per section (×200 magnification). Both areas were measured and quantitatively compared as described previously (28). All quantifications specifically targeted the lung periphery and excluded larger vessels and airways.

### Immunofluorescence

Detection of PDGFRα (sc-12911; Santa Cruz Biology; 1:200), TGF-β (ab63399; Abcam; 1:400), Collagen-1 (ab34710; Abcam; 1:150), αSMA (14–9760-82; Thermo Fisher; 1:200), and Caspase 3 (ab13847; Abcam; 1:200) was done using immunofluorescence (4-5 individual animals) as previously described (24, 26, 27). Images were acquired using an AXIO Imager M2 (Zeiss, Germany). Quantitative assessment of signal intensity was done in four FOV per section (×400 magnification) and normalized to 100 nuclei using the Bioquant software.

### Immunohistochemistry

Sections from 4 animals per group were incubated with peroxidase blocking reagent (31642; Sigma-Aldrich), normal goat serum, primary antibodies [anti-F4/80; ab6640 (Abcam); 1:400], and biotinylated goat anti-rat secondary antibody (Santa Cruz Biotechnology; 1:200). Visualization with streptavidin-horseradish peroxidase (HRP) (GERPN1231; Merck) and diaminobenzidine was completed by counterstaining hematoxylin (Richard-Allan Scientific) and Dako mounting medium (S3023, Agilent). The number of F4/80-positive cells per alveoli was quantified by light microscopy in 6 FOV per slide (×400 magnification) using FIJI (29).

### In Vitro Experiments

Mouse myofibroblasts (MFBs) were extracted and cultured from the lungs of 5–7-day-old C57BL6 mice as described previously (27). Cultured cells were then incubated with TGF-β (5 ng/mL) (100-21, PeproTech) or a combination of TGF-β (5 ng/mL), 10^−6^ M all-trans retinoic acid (ATRA; R2625, Sigma-Aldrich), and 10^−7^ M 1,25-dihydroxy vitamin D3 (VitD) (D1530, Sigma- Aldrich) for 40 h as previously described (30). At the end of the experiment, cells were lysed with radioimmunoprecipitation assay buffer (RIPA buffer) and processed for immunoblot analysis (25). Restore Western Blot stripping buffers (21059, Thermo Fisher) were used to remove the antibodies from the membranes to allow reprobing. Antibodies included PDGFRα (338, Santa Cruz Biotechnology), pERK (4370, Cell Signaling Technologies), pSMAD2-3 (PA5-99378, Thermo Fisher), vitamin D receptor (MA1-710, Thermo Fisher), and retinoid X receptor (5388, Cell Signaling).

### Statistical Analysis

Statistical analysis was performed using the Prism 9 software package (GraphPad, San Diego, CA). For multiple testing, we used a two-way analysis of variance (ANOVA) with Bonferroni correction. For comparisons of in vitro groups/parameters, we used one-way ANOVA with Dunnett’s correction. Results are given as means ± SD, and the number of experiments is presented in the figure legends.

## RESULTS

### Prenatal Vitamin D Supplementation Decreased Monocyte/Macrophage Presence in the Developing Lung Undergoing Postnatal Injury

**Figure 1.**
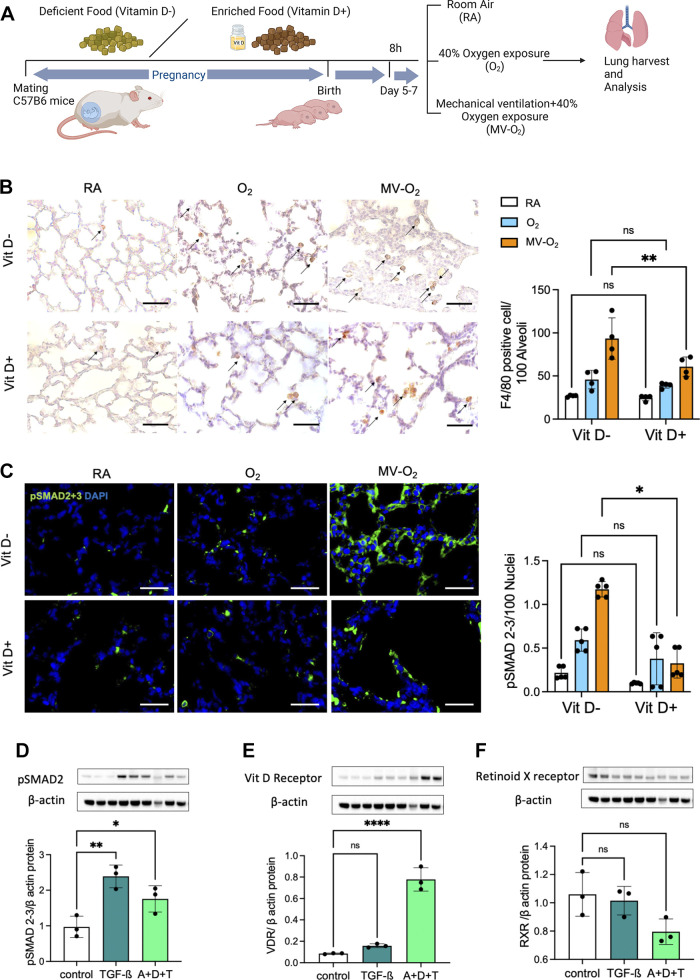
*A*: in a preclinical mouse model of bronchopulmonary dysplasia (BPD), mice were subjected to vitamin D-enriched (VitD+) or -deprived (VitD−) food during pregnancy followed by the postnatal exposure to room air (RA), oxygen (O_2_, Fi_O_2__ = 0.4)_,_ with or without mechanical ventilation (MV). *B*: immunohistochemistry (IHC) revealed a significant increase in the number of F4/80-positive monocytes/macrophages in the lungs undergoing postnatal injury, which was ameliorated after prenatal vitamin D supplementation (VitD+). The scale bar is 100 μm. *C*: subsequently, immunofluorescence (IF) showed a significant reduction of pSMAD2/3 expression in the neonatal lung exposed to postnatal injury (MV) after VitD+ food during pregnancy. The scale bars are 50 μm. *D*: immunoblot analysis demonstrated a significant reduction in pSMAD2/3 expression in vitamin D-treated primary neonatal mouse lung fibroblasts after stimulation with transforming growth factor-beta (TGF-β1; 5 ng/mL). *E*: immunoblot analysis furthermore confirmed the upregulation of vitamin D receptor (VDR) expression after treatment [10^−7^ M vitamin D supplement (D) + 10^−6^ M vitamin A cotreatment supplement (A) + 5 ng/mL TGF-β1 (T)]. *F*: in contrast, no significant differences in retinoid X receptor (RXR) expression were observed. Data are given as means ± SD. *P < 0.05, **P < 0.01, ****P < 0.0001, two-way analysis of variance (ANOVA) with Bonferroni correction; *n* = 4-5 individual mice. Identical β-actin due to immunoblot reprobing (*D, E, F*) [Image created with BioRender.com and published with permission].

We studied the effect of differences in prenatal vitamin D supplementation (from the onset of mating until the end of gestation) using a vitamin D-enriched (1,500 IU/kg) or -deprived (<10 IU/kg) diet on the pulmonary response to postnatal injury, i.e., O_2_ and MV-O_2_ exposure in a preclinical BPD mouse model (Fig. 1*A*). With either diet, we observed no differences in body weight (*P* = 0.702) or lung volume (*P* = 0.842) (Supplemental Fig. S1, *A* and *B*). As postnatal injury is known to trigger a sustained inflammatory response in the neonatal lung that is causally related to BPD development (31), we assessed the injury-induced presence of F4/80-positive monocytes/macrophages in the neonatal mouse lung after postnatal exposure to O_2_ and MV-O_2_. In line with our previous studies (24), MV-O_2_ provoked the recruitment of monocytes/macrophages to the lung periphery. In neonates that had received VitD+ food during pregnancy, we observed a 1.5-fold reduction of lung F4/80-positive monocytes/macrophages under MV-O_2_ when compared with pups born after VitD− food during gestation (Fig. 1*B*). The decreased number of F4/80-positive monocytes/macrophages upon vitamin D supplementation during pregnancy was accompanied by an up to threefold reduction in pulmonary TGF-β pathway activation, i.e., pSMAD 2/3 expression in lungs exposed to MV-O_2_ (Fig. 1*C*). In vitro, we successfully recapitulated the reduction in TGF-β activation by 10^−7^ M vitamin D treatment in primary neonatal mouse lung fibroblasts (Fig. 1*D*), together with an upregulation of the vitamin D receptor following the treatment (Fig. 1*E*). However, we did not observe any significant difference in the retinoid X receptor (RXR) expression under these in vitro conditions (Fig. 1*F*).

### Prenatal Vitamin D Supplementation Ameliorated Injury-Induced Lung Matrix Remodeling

**Figure 2.**
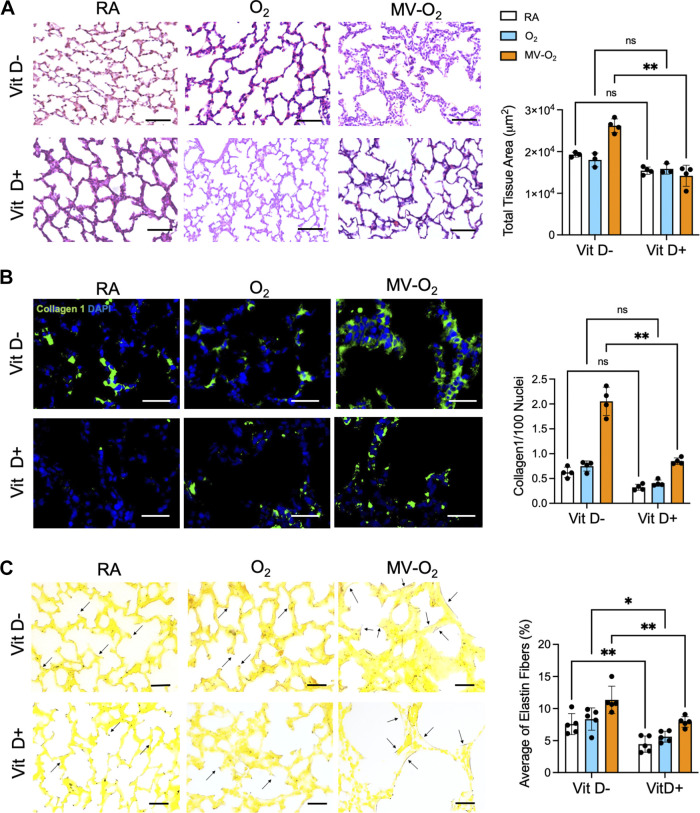
*A*: analysis of markers indicating lung matrix remodeling demonstrated a reduction in total lung tissue area in neonatal mouse lungs under room air (RA) conditions and when undergoing oxygen exposure with mechanical ventilation (MV-O_2_) after vitamin D-enriched (VitD+) food during pregnancy as compared with neonatal mice that received (VitD−) deprived food. The scale bars are 100 μm. *B*: immunofluorescence (IF) staining for type I collagen (green) revealed a decreased expression in mice receiving VitD+ food under RA conditions and undergoing oxygen (O_2_) exposure with or without MV. The scale bar is 50 μm. *C*: Hart’s stain analysis demonstrated a decreased expression of insoluble cross-linked elastic fibers in neonatal mice after a prenatal VitD+ diet and a decrease of its pathological deposition in response to MV-O_2_ in these mice. The scale bars are 100 μm. Data are means ± SD. *P < 0.05, **P < 0.01, two-way analysis of variance (ANOVA) with Bonferroni correction; *n* = 4-5 individual mice.

Lung matrix remodeling is a crucial characteristic of BPD development and is majorly driven by pulmonary inflammation (46). In neonatal mice that received VitD+ food during gestation, interstitial thickening of the gas exchange area in response to postnatal injury, i.e., O_2_ and MV-O_2_ exposure was significantly reduced as compared with neonatal mice that had received VitD− food during gestation (Fig. 2*A*). Underlying this finding, type I collagen deposition in the lung periphery was significantly less in neonatal mice born after VitD supplementation (VitD+) during gestation when compared with VitD− pups (Fig. 2*B*), together with a significantly reduced pathological deposition of mature elastic fibers in response to MV-O_2_, previously shown to characterize lung injury (32) (Fig. 2*C*). We did not observe significant differences in radial alveolar counts (RACs) or alveolar area when comparing the two different prenatal diet regimens (VitD+ vs. VitD−) for the different injury conditions. With lung volume showing no significant differences between study groups (*P* = 0.842) (Supplemental Fig. S1), a comparable degree of lung inflation can be assumed.

### Reduced Presence of Proproliferative Neonatal Lung Fibroblasts following Prenatal Vitamin D Supplementation

**Figure 3.**
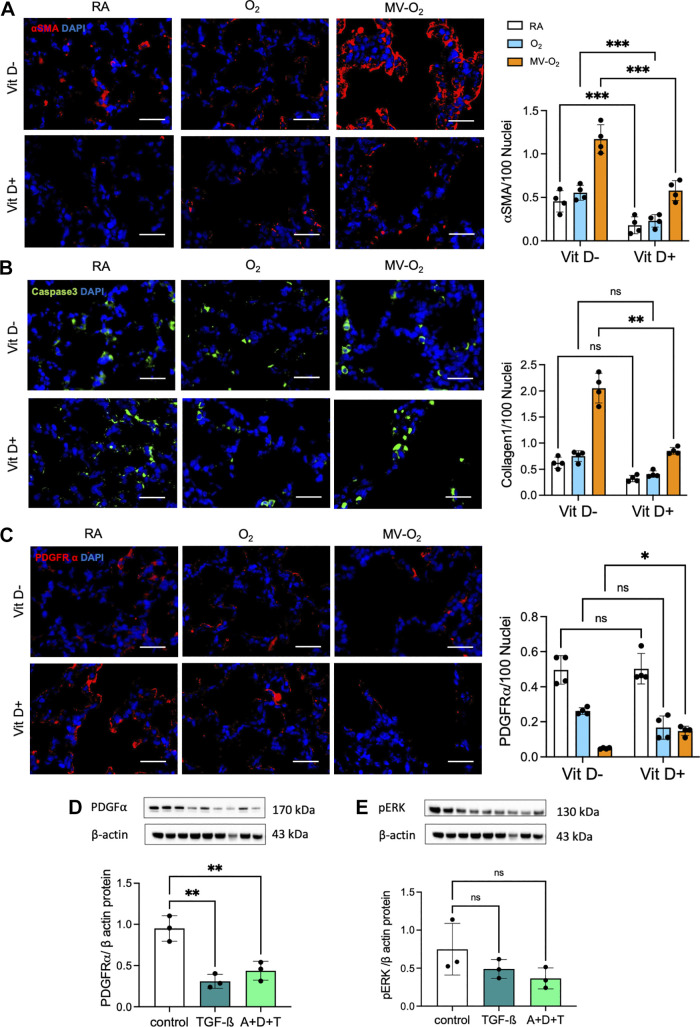
*A*: immunofluorescence (IF) staining of alpha-smooth muscle actin (α-SMA; red) showed a significant decrease in neonates that received vitamin D-enriched (VitD+) food during pregnancy under room air (RA) conditions and after oxygen (O_2_, Fi_O_2__ = 0.4) exposure with and without mechanical ventilation (MV) as compared with neonates that received vitamin D-deprived (VitD−) food. The scale bars are 50 μm. *B*: the effect of prenatal VitD+ supplementation on fibroblast activation is accompanied by a reduced rate of apoptosis in mouse lungs undergoing postnatal injury, i.e., MV-O_2._ The scale bars are 50 μm. *C*: IF staining for platelet-derived growth factor alpha (PDGFRα, red) showed preserved expression in neonatal mouse lungs after MV-O_2_ exposure in neonatal mice after prenatal supplementation (VitD+) as compared with mice that received VitD− food during pregnancy. The scale bars are 50 μm. *D*: in vitro immunoblot analysis of primary neonatal mouse lung fibroblasts confirmed the preservation of PDGFRα expression in cells exposed to TGF-β1 (5 ng/mL) when treated with vitamin D [10^−7^ M vitamin D supplement (D) + 10^−6^ M vitamin A cotreatment supplement (A) + 5 ng/mL TGF-β (T)]. *E*: immunoblot analysis in vitro did not show a significant difference in pERK expression as an independent apoptosis pathway. Data are given as means ± SD. *P < 0.05, **P < 0.01, ***P < 0.001, two-way analysis of variance (ANOVA) with Bonferroni correction; *n* = 4 individual mice. Identical β-actin due to immunoblot reprobing (*D* and *E*).

In line with the decrease in matrix remodeling, prenatal VitD+ food resulted in the reduced presence of activated, i.e., αSMA-positive pulmonary fibroblasts in the gas exchange area of the neonatal mouse lung undergoing postnatal O_2_ and MV-O_2_ exposure (Fig. 3*A*), known as the important indicator of lung fibroblast-driven matrix remodeling (32). In addition, we observed a significant reduction in lung apoptosis as indicated by caspase 3 expression in neonatal lungs from the VitD+ diet group (Fig. 3*B*). Highlighting a critical driver of alveolar septation (32), VitD+ food during gestation resulted in the preserved expression of PDGFRα in the gas exchange area of the neonatal lung undergoing O_2_ exposure or MV-O_2_ (Fig. 3*C*).

In vitro, we successfully recapitulated the stimulation of PDGFRα expression in primary neonatal lung fibroblasts by vitamin D treatment (10^−7^ M) next to the reduction of TGF-β activation in the treated cells (Fig. 3*D*). To evaluate the effect of vitamin D on apoptosis, we assessed protein kinase RNA-like ER kinase (pERK) expression levels in primary neonatal lung fibroblasts but did not detect significant changes in this independent apoptosis pathway (Fig. 3*E*).

## DISCUSSION

To address the potential impact of vitamin D supplementation on lung health during development (32, 33), we evaluated the effect of prenatal vitamin D on the postnatal pulmonary response to injury including immunomodulatory functions. Here, we successfully demonstrated a decrease in the number of monocytes/macrophages in the developing lung upon postnatal injury when VitD+ food was provided during pregnancy, together with a reduction of pulmonary TGF-β activation, typically observed after O_2_ and MV exposure (28). Vitamin D has been studied for its modulation of pulmonary inflammation (34) and its effects on innate immune defense responses, including critical functions of monocyte and alveolar macrophages (35). As monocytes and macrophages have been identified for their crucial role in BPD development (47), we targeted their presence in the developing lung undergoing postnatal injury while modifying prenatal vitamin D supplementation. As main drivers of lung inflammation and structural injury, monocytes/macrophages in the neonatal lung are likely affected directly by differing vitamin D levels due to their expression of the vitamin D receptor, enabling them to respond to the active vitamin D metabolite 1,25(OH)D_2_ (36). The 1,25(OH)D_2_-triggered response is dominated by the differentiation of monocytes toward a macrophage-like phenotype, thereby impacting on pulmonary innate immune responses. As different types of pulmonary macrophages are relevant for lung development and disease, it will be crucial to investigate the impact of vitamin D supplementation in this regard in future studies (37, 38)

In line with the reduced activation of the TGF-β pathway, we observed a reduction of its proapoptotic and profibrotic activities upon prenatal vitamin D supplementation as exemplified by a reduced number of α-SMA-positive lung fibroblasts. Subsequently, the postnatally injured gas exchange area was characterized by ameliorated interstitial thickening accompanied by reduced collagen deposition and pathological elastin deposition in pups exposed to vitamin D supplementation during pregnancy, all described as characteristics of postnatal lung injury (26, 27, 39, 40). Together with the preserved number of PDGFRα-positive cells in the injured gas exchange area, the changes in apoptosis, stalled developmental progress and remodeling occurring in the developing mouse lung that postnatally rapidly transitions through alveolarization in the first week of life, likely affect alveolarization as well as later regeneration potential of the postnatally injured lung. The findings furthermore reflect on direct effects of vitamin D on alveolar signaling pathways (41), including PDGF-dependent fibroblast proliferation (30), next to the immune-modulating effects outlined earlier. Our results support the influence of vitamin D on the vulnerability of the lung toward postnatal exposure to O_2_ and/or MV. Although mice models are the most commonly used model organisms in disease research and are able to gain valuable insight into human pathology, there are, however, important differences including the regulation of the vitamin D receptor gene and its expression (42). Therefore, confirmation of our data in human primary cells and clinical datasets is needed next to future studies by increasing group size, duration of ventilation, addressing other potential limitations such as sex-specific responses as well as the additional effect of prolonged vitamin D insufficiency or deficiency after birth. Moreover, other studies revealed the importance of sex-dependent effects of vitamin D deficiency in airway disease (43). In addition, expression of the vitamin D receptor on B-, T- and antigen-presenting cells (44) highlights the need to investigate adaptive immune responses in the injured neonatal lung both short and longer term. The lack of vitamin D serum concentration in neonatal mice due to the fact that sufficient amounts of blood in these mice at birth can only be obtained by (fatal) heart puncture was balanced by the adaptation of a diet strategy during pregnancy that had been published as safe and efficient before (25). Not only were previous studies able to reach sufficient 25(OH)D3 levels by comparable supplementation doses as used in our VitD+ diet (45) but also duration and dose applied in the vitamin D3-deprived group (VitD−) likely resulted in insufficient plasma levels when compared with previously published data (12, 16).

To mimic the most prevalent clinical condition, i.e., vitamin D insufficiency affecting up to 70% of pregnant women (23) and to demonstrate that “subclinical” vitamin D insufficiency during pregnancy is able to impact neonatal outcome, we successfully demonstrated a pulmonary phenotype dependent on vitamin D supplementation, while assuring clinical “silence” of the applied diet in our animal model regarding the health status of the dams and the offspring, i.e., no differences in birth weight or lung volume.

In summary, we successfully demonstrated that prenatal vitamin D supplementation reduced lung inflammation and subsequent events such as remodeling of the gas exchange area in neonatal mice exposed to postnatal lung injury. We believe that our data that delineated the effect of prenatal vitamin D supply on the vulnerability of the neonatal lung toward postnatal injury support the notion to fight vitamin D insufficiency in pregnant women and underline the importance to investigate the modulation of the inflammatory response by vitamin D further to develop therapeutic and preventive strategies.

## DATA AVAILABILITY

All data are available in the main text or the supplemental material.

## SUPPLEMENTAL MATERIAL

10.6084/m9.figshare.22903541.v1 Supplemental Fig. S1: https://doi.org/10.6084/m9.figshare.22903541.v1.

## GRANTS

The present study was supported by the Young Investigator Grant NWG VH-NG-829 by the Helmholtz Foundation and the Helmholtz Zentrum München, Germany, the German Center for Lung Research (DZL, German Ministry of Education and Health (BMBF)) as well as the Research Training Group Targets in Toxicology (GRK2338) of the German Science and Research Organization (DFG). Additional financial support was provided by the Stiftung AtemWeg (LSS AIRR).

## DISCLOSURES

No conflicts of interest, financial or otherwise, are declared by the authors.

## AUTHOR CONTRIBUTIONS

M.W. and A.H. conceived and designed research; J.W., Mi.H., P.O., M.K., and N.K., performed experiments; J.W., M.H., and A.H. analyzed data; J.W., M.H., and A.H. interpreted results of experiments; J.W., M.H., and A.H. prepared figures; J.W., M.H., and A.H. drafted manuscript; J.W., M.H., M.W., and A.H. edited and revised manuscript; J.W., M.H., P.O., M.K., N.K., Mi.H., M.W., and A.H. approved final version of manuscript.
